# Paratracheal pressure reduces the incidence of moderate-to-severe coughing during endoscopic esophageal iodine staining: a prospective, randomized controlled trial

**DOI:** 10.1186/s12871-026-03644-y

**Published:** 2026-02-02

**Authors:** Cheng Zhang, Yingying Lu, Tieli Xie, Chunxue Zhang, Jia Yang, He Ma, Zhuqing Rao

**Affiliations:** 1 Department of Anesthesiology, the First Affiliated Hospital with Nanjing Medical University, Nanjing, Jiangsu China; 2 Department of Anesthesiology, Peixian People’s Hospital, Xuzhou, Jiangsu China

**Keywords:** Paratracheal pressure, Esophageal iodine staining, Endoscopy, Cough prevention, Anesthesiologist-assisted

## Abstract

**Background:**

Endoscopic iodine staining is a crucial technique for diagnosing esophageal cancer and precursor lesions. However, it carries the risk of adverse events such as pharyngeal discomfort, coughing, and aspiration. The study aimed to investigate the effectiveness of anesthesiologist-assisted paratracheal pressure in reducing the incidence of coughing during this procedure.

**Methods:**

Patients were randomly allocated to two groups. In the treatment group (paratracheal pressure), when the endoscope reached approximately 20 cm from the incisors, the anesthesiologist performed paratracheal pressure just before iodine spraying and maintained it for 5 s after spraying ceased. The endoscopist then observed the staining pattern. The pressure was reapplied during saline rinsing and released 5 s after rinsing completion. The control group (no paratracheal pressure) underwent the standard procedure without paratracheal pressure. The primary outcome was the incidence of moderate-to-severe coughing during the procedure.

**Results:**

The incidence of moderate-to-severe cough was significantly lower in the treatment group than in the control group (7.50% (3/40) vs. 32.50% (13/40); risk difference = − 25.00%, 95% confidence interval (CI): − 40.48% to − 9.52%). This association remained statistically significant after multivariable adjustment (adjusted odds ratio = 0.11, 95%CI: 0.02 to 0.55; *P* = 0.007). At 2 min post-compression, the treatment group also exhibited significantly lower mean arterial pressure (MAP: 88.05 ± 5.82 vs. 93.83 ± 5.07 mmHg) and heart rate (HR: 68.70 ± 7.91 vs. 73.03 ± 10.39 bpm). These differences were further confirmed by analysis of covariance.

**Conclusions:**

Anesthesiologist-assisted paratracheal pressure effectively reduces the incidence of moderate-to-severe coughing and pharyngeal discomfort during endoscopic iodine staining, while also promoting greater hemodynamic stability.

**Trial registration:**

ChiCTR2400088832.

**Supplementary Information:**

The online version contains supplementary material available at 10.1186/s12871-026-03644-y.

## Introduction

Endoscopic esophageal iodine staining is a widely used technique for the screening of esophageal cancer, playing a crucial role in improving patient survival rates [1, 2]. During this procedure, iodine solution is sprayed onto the esophageal mucosa, highlighting lesions through a distinct color contrast with normal tissue, thereby significantly enhancing the detection rate of early-stage abnormalities [3]. This method is valued in clinical practice for its simplicity, cost-effectiveness, and diagnostic efficacy. Nevertheless, the procedure carries certain risks and challenges. Coughing is one of the most common adverse reactions during endoscopic iodine staining. It is typically triggered when the iodine solution inadvertently enters the airway, irritating the pharyngeal and tracheal mucosa and eliciting a reflex cough [4]. This reaction not only compromises patient comfort and procedural accuracy but may also lead to more serious complications.

Therefore, effectively preventing cough during endoscopic iodine staining has become a pressing issue in clinical practice. With advancements in anesthesia and airway management techniques, paratracheal pressure has emerged as a promising method for airway control. This technique involves applying pressure to the paratracheal area above the clavicle, which can effectively occlude the upper esophagus [5, 6]. Nonetheless, there is a scarcity of studies investigating the application of paratracheal pressure specifically for preventing cough during endoscopic iodine staining, and its efficacy and safety require further validation.

The randomized controlled trial (RCT) aimed to evaluate the effectiveness of anesthesiologist-assisted paratracheal pressure in preventing cough during endoscopic esophageal iodine.

## Methods

### Study design and ethics

This single-center, randomized controlled trial was conducted with blinding of participants. The study protocol received approval from the Ethics Review Committee of Peixian People’s Hospital, China (approval number: 2024(15)). The trial was registered at the Chinese Clinical Trial Registry (Clinical trial number: ChiCTR2400088832 ||http://www.chictr.org.cn/||August 27, 2024). The research adhered to the ethical principles outlined in the 1964 Helsinki Declaration and its subsequent revisions, and all participants provided written informed consent prior to enrollment.

### Participants

Eligible participants were randomly assigned in a 1:1 ratio to either the treatment group (paratracheal pressure) or the control group (no paratracheal pressure). The following were the inclusion criteria: (1) scheduled for endoscopic esophageal iodine staining; (2) planned for intravenous anesthesia; (3) aged 18 to 65 years, having provided signed informed consent for anesthesia; (4) clear consciousness postoperatively without cognitive impairment; (5) body mass index (BMI) between 18.5 and 28 kg/m²; and (6) ASA physical status classification of I to III. The exclusion criteria were patients: (1) patients at high risk for pulmonary aspiration; (2) a history of neck trauma or severe cervical spondylosis; (3) known allergy to iodine or general anesthetics. Criteria for withdrawal from the study were: (1) voluntary withdrawal by the patient; (2) loss to follow-up; (3) decision by the investigators due to reasons including, but not limited to, poor patient compliance, the occurrence of significant complications, or serious adverse events.

### Randomization and blinding

A computer-generated simple randomization sequence with a 1:1 allocation ratio was prepared by an independent researcher not involved in the trial’s execution, follow-up, or data analysis. To ensure allocation concealment, the assignments were sealed in sequentially numbered, opaque envelopes. These envelopes were opened by the attending clinicians in the operating room immediately prior to the procedure. All participants were blinded to treatment assignment. Due to the nature of the intervention, the anesthesiologist delivering the paratracheal pressure also assessed the primary outcome and was therefore unblinded. In contrast, data for exploratory outcomes—including postoperative discomfort, fever, and laboratory markers—were collected by independent research staff who remained blinded to group allocation throughout the study period.

### Trial procedure

All endoscopic procedures were performed using a Fujifilm ELUXEO 7,000 system (Fujifilm Corporation, Tokyo, Japan) by a single experienced associate chief physician in gastroenterology. Prior to participant enrollment, the senior anesthesiologist responsible for delivering the paratracheal pressure maneuver completed standardized training, which included repeated practice sessions using an electronic force gauge with real-time feedback until consistent application of the target 30 N pressure was achieved [6, 7]. The same anesthesiologist performed all interventions throughout the trial, ensuring consistent procedural proficiency. Eligible patients with normal preoperative electrocardiograms and an ASA physical status classification of I–III provided written informed consent prior to participation.

Ten minutes before the procedure, patients orally received 10 mL of dyclonine hydrochloride mucilage (10 mL: 0.1 g; Yangtze River Pharmaceutical Group, Batch No.: 23101311) and 0.3 g of simethicone powder (5 g: 0.3 g/bottle; Zigong Honghe Pharmaceutical Co., Ltd., Batch No.: 240211) to reduce secretions and improve visualization.

Patients were placed in the left lateral position. Nasal oxygen was delivered at 5 L/min, and continuous monitoring was established using a BeneVision N15 patient monitor (Mindray, Shenzhen, China). Monitoring included non-invasive blood pressure (NIBP), electrocardiography (ECG), and peripheral oxygen saturation (SpO_2_). An intravenous catheter was placed in the right upper limb, and NIBP was recorded every 3 min during the procedure.

Anesthesia was induced with remifentanil (0.2 µg/kg), remimazolam tosilate (0.1 mg/kg), and propofol (1.5–2.0 mg/kg). Additional boluses of propofol were administered intraoperatively as needed to maintain adequate sedation. If systolic blood pressure decreased by more than 30% from baseline, phenylephrine (50–100 µg) was administered incrementally. After loss of the eyelash reflex, standard esophagogastroduodenoscopy was initiated. Upon reaching the gastroesophageal junction, a spray catheter was advanced through the biopsy channel. Under direct visualization, 15 mL of 1.2% Lugol’s iodine solution was uniformly sprayed from the distal to proximal esophagus.

#### Treatment group (paratracheal pressure)

When the iodine spray reached approximately 20 cm from the incisors, the anesthesiologist applied paratracheal pressure using the index and middle fingers of the right hand, placed adjacent to the trachea just above the left clavicle and medial to the sternocleidomastoid muscle. A target force of 30 N was applied and maintained for 5 s after completion of staining, then released. This maneuver was repeated during the subsequent 50 mL saline rinse and held for 5 s after rinse completion. The anesthesiologist underwent standardized training using an electronic force gauge to ensure accurate and consistent pressure application, with refresher training conducted every two months throughout the study period.

#### Control group (no paratracheal pressure)

Patients underwent identical endoscopic and anesthetic procedures without application of paratracheal pressure.

### Outcomes

The primary outcome was the incidence of moderate-to-severe coughing during the endoscopic iodine staining procedure, assessed using the Minogue Grade [8]. Coughing was classified as follows: Grade 1: single cough; Grade 2: repeated coughing lasting ≤ 5 s; Grade 3: persistent coughing lasting > 5 s. Grades 2 and 3 were considered positive events for the primary outcome (with Grade 3 representing severe cough); Grade 1 was not included.

Secondary outcomes included two hemodynamic parameters: mean arterial pressure (MAP) and heart rate (HR), measured at baseline (immediately before paratracheal pressure, T0) and 2 minutes after pressure (T1). Exploratory outcomes, assessed within 24 hours after the procedure, included: (1) intraoperative and postoperative hypoxemia (defined as SpO₂ < 90% for ≥ 30 seconds during endoscopy or within 30 minutes after completion, respectively) [9]; (2) pharyngeal, chest, and abdominal discomfort, evaluated separately using a 10-cm visual analog scale (VAS; 0 = “no discomfort”, 10 =“worst imaginable discomfort”) [10–12], with a score of ≥ 3 at any site considered clinically significant; (3) postoperative fever (oral or tympanic temperature ≥ 38.5 °C); (4) occurrence of any severe cough episode within 24 h post-procedure; and (5) Levels of C-reactive protein, white blood counts, neutrophil percentage, lymphocyte percentage, and platelet counts.

### Sample size calculation

The sample size was calculated based on preliminary data, which indicated coughing incidences of 8.5% in the treatment group and 36.3% in the control group. Using a two-sided Z-test for two proportions (pooled variance) in PASS 2021, with a significance level (α) of 0.05 and a power (1-β) of 0.9, a minimum of 40 patients per group was required to detect this difference.

### Statistical analysis

Continuous variables were summarized as mean ± standard deviation (Mean ± SD) if they followed a normal distribution. For non-normally distributed data, results were reported as median and interquartile range [M (P25, P75)]. Categorical variables were presented as counts (n) and percentages (%). In addition to absolute proportions, risk ratio (RR), risk difference (RD), and their corresponding 95% confidence intervals (95% CI) were reported. To account for potential baseline imbalances and enhance causal interpretation, we further performed a multivariable logistic regression analysis adjusting for clinically relevant covariates: age, sex, BMI, smoking history, and ASA physical status classification. Secondary outcomes, measured at 2 min post-compression, were compared between groups using analysis of covariance (ANCOVA), with baseline (pre-procedural) MAP or HR (as appropriate) and age entered as covariates. Results are presented as adjusted between-group mean differences (MDs) with 95% CIs and corresponding *P* values. For exploratory outcomes—including intra- and postoperative hypoxemia, site-specific discomfort (pharyngeal, chest, abdominal), postoperative fever, 24-hour cough episodes, and inflammatory/hematologic markers (C-reactive protein, white blood cell counts, neutrophil percentage, lymphocyte percentage, and platelet counts)—no formal hypothesis testing was conducted. To control the familywise Type I error rate, only descriptive statistics (e.g., proportions, medians with interquartile ranges, or means with standard deviations, as appropriate) are presented without *p*-values.

Statistical significance was defined as a two-sided *p*-value less than 0.05. All statistical analyses were performed using SPSS software (version 27.0).

## Results

### Peri-, Intra- and postoperative characteristics

**Fig. 1:**
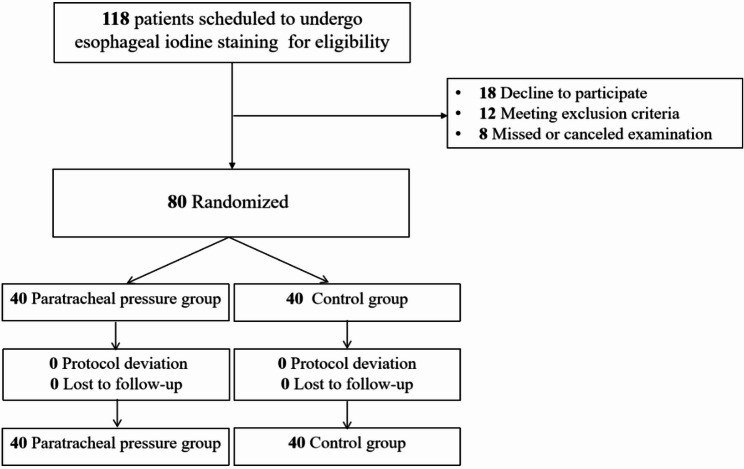
CONSORT 2010 flow diagram

Patients scheduled for esophageal iodine staining were enrolled between September 2024 and January 2025 and assessed for eligibility. Of the 118 individuals assessed, 38 did not proceed to randomization: 18 declined to participate, 12 met exclusion criteria, and 8 missed or canceled their examination. A total of 80 eligible patients were recruited and randomly allocated to the treatment group (*n* = 40) or control group (*n* = 40). All participants completed the assigned intervention and were included in the final analysis. No participants were lost to follow-up, and there were no protocol violations or serious adverse events in either group. The flow of participants is illustrated in the CONSORT diagram (Fig. 1) [13, 14].

The two groups had comparable baseline demographics, comorbidities (e.g., smoking history, hypertension, diabetes, coronary heart disease), and laboratory findings (including C-reactive protein, white blood cell counts, neutrophil and lymphocyte percentages, and platelet counts), as shown in Table 1.

**Table 1:** Baseline characteristics

	Treatment group	Control group
General characteristics		
Age, year, Mean ± SD	51.62 ± 9.25	51.38 ± 7.29
Sex		
Male, *n*(%)	15(37.50)	17 (42.50)
Female, *n*(%)	25(62.50)	23 (57.50)
Height, cm, Mean ± SD	166.32 ± 10.11	168.47 ± 11.18
Weight, kg, Mean ± SD	66.50 ± 12.50	68.47 ± 13.39
BMI, kg/m², Mean ± SD	23.72 ± 2.34	23.79 ± 2.18
ASA, Ⅰ/Ⅱ/Ⅲ (*n*)	11/26/3	12/24/4
Comorbidities		
Smoking history, *n* (%)	12(30.00)	14(35.00)
Hypertension, *n* (%)	13(32.50)	11(27.50)
Diabetes, *n* (%)	7(17.50)	5(12.50)
Coronary heart disease, *n* (%)	6(15.00)	9(22.50)
Procedure time, min, Mean ± SD	8.25 ± 1.03	8.03 ± 1.27
Preoperative laboratory tests		
C-reactive protein, mg/L, Mean ± SD	3.74 ± 1.54	3.73 ± 1.52
White blood cell counts, 10^^9^/L, Mean ± SD	6.16 ± 1.53	6.21 ± 1.51
Neutrophils, %, Mean ± SD	59.90 ± 10.43	60.00 ± 8.92
Lymphocytes, %, Mean ± SD	30.82 ± 8.92	30.90 ± 7.69
Platelet counts, 10^^9^/L, Mean ± SD	215.80 ± 55.65	214.18 ± 45.16

Treatment group: paratracheal pressure; control group: no paratracheal pressure

*BMI* body mass index, *ASA* American society of anesthesiologists

### Primary outcome

 The overall incidence of moderate-to-severe coughing among all participants was 20.00% (16/80). In the paratracheal pressure group, the incidence was 7.50% (3/40), compared with 32.50% (13/40) in the control group (Table 2). The RR of moderate-to-severe coughing in the treatment group was 0.23 (95% CI: 0.07 to 0.75), corresponding to an absolute RD of − 25.00 percentage points (95% CI: − 40.48 to − 9.52; Table 2). In a multivariable logistic regression model adjusting for age, sex, BMI, smoking status, and ASA, the odds of moderate-to-severe coughing were significantly lower in the treatment group (adjusted OR = 0.11; 95% CI: 0.02 to 0.55; *P* = 0.007) (Table 2).

**Table 2:** Primary and secondary outcomes

Outcomes	Treatment group	Control group	Effect Estimate (95% CI)	*P*
Primary Outcome				
Moderate-to-severe coughing, n(%)	3(7.50)	13(32.50)	RR = 0.23 (0.07 to 0.75)	
			RD = -25.00% (-40.48% to -9.52%)	
			aOR = 0.11 (0.02 to 0.55)	0.007
Secondary Outcomes				
MAP, mmHg, Mean ± SD	88.05 ± 5.82	93.83 ± 5.07	aMD =–6.12 (–8.59 to − 3.64)	< 0.001
HR, bpm, Mean ± SD	68.70 ± 7.91	73.03 ± 10.39	aMD =–3.53 (–6.65 to − 0.41)	0.027

*MAP* mean arterial pressure, *HR* heart rate, *RR* Risk ratio, *RD* Risk difference, *95% CI* confidence interval, *aOR* adjusted odds ratio, *aMD* adjusted mean difference

**Table 3:** Exploratory outcomes

	Treatment group	Control group
Intraoperative hypoxemia, *n*(%)	4(10.00)	10(25.00)
Postoperative hypoxemia, *n* (%)	0(0.00)	2(5.00)
Sore throat or pharyngeal discomfort, *n* (%)	5(12.50)	21(52.50)
Chest or abdominal discomfort, *n* (%)	22(55.00)	18(45.00)
Postoperative fever, *n* (%)	0(0.00)	1(2.50)
Severe coughing, *n* (%)	0(0.00)	3(7.50)
C-reactive protein, mg/L, Mean ± SD	3.83 ± 1.33	4.00 ± 1.69
White blood cell counts, 10^^9^/L, Mean ± SD	6.44 ± 1.41	6.64 ± 1.55
Neutrophils, %, Mean ± SD	60.10 ± 10.24	60.77 ± 8.99
Lymphocytes, %, Mean ± SD	30.36 ± 9.09	30.40 ± 7.35
Platelet counts, 10^^9^/L, Mean ± SD	218.43 ± 51.19	221.68 ± 49.80

### Secondary and exploratory outcomes

Patients receiving paratracheal pressure exhibited significantly lower MAP and HR at 2 min post-compression compared to the control group. The unadjusted values were: MAP, 88.05 ± 5.82 vs. 93.83 ± 5.07 mmHg; HR, 68.70 ± 7.91 vs. 73.03 ± 10.39 bpm. After adjustment for baseline values and age, the treatment group showed a mean reduction in MAP of 6.12 mmHg (95% CI: − 8.59 to − 3.64; *P* < 0.001, Table 2) and in HR of 3.53 bpm (95% CI: − 6.65 to − 0.41; *P* = 0.027, Table 2). Furthermore, the incidence of sore throat or pharyngeal discomfort was markedly lower in the treatment group (12.50%) than in the control group (52.50%) in Table 3. A similar reduction was observed for intraoperative hypoxemia (10.00% vs. 25.00%). Additionally, no cases of postoperative hypoxemia, postoperative fever, or severe coughing were reported in the treatment group, whereas these events occurred in a small number of patients in the control group. In contrast, the values for C-reactive protein, white blood cell counts, neutrophil percentage, lymphocyte percentage, and platelet counts appeared numerically similar between the two groups.

## Discussion

In this study, we demonstrate that anesthesiologist-assisted paratracheal pressure contributes to a reduced incidence of moderate-to-severe coughing and pharyngeal discomfort during endoscopic iodine staining, along with improved hemodynamic stability.

Paratracheal pressure demonstrates remarkable superiority in preventing gastroesophageal reflux and optimizing airway management [6]. The trachea and esophagus are closely aligned in the cervical region, with the trachea situated anteriorly and composed of rigid cartilaginous rings, whereas the esophagus lacks such supportive structures and is more pliable. Based on ultrasonographic anatomy, the cervical esophagus deviates to the left from the midline as it descends into the thorax. Application of paratracheal pressure above the left clavicle compresses the anterior wall of the esophagus, effectively closing its lumen, which shows a significant change in esophageal cavity dimensions before and after compression. Consequently, paratracheal pressure has been proposed for occluding the upper esophagus within the paratracheal area to prevent gastric reflux.

Multiple studies have highlighted the efficacy of paratracheal pressure in reducing gastroesophageal reflux and enhancing airway management. Specifically, in patients undergoing general anesthesia, paratracheal pressure facilitates easier placement of the i-gel™ supraglottic airway device compared to cricoid pressure, with higher insertion success rates, shorter insertion times, lower resistance during insertion, and minimal impact on ventilation [15]. Furthermore, in adult patients under general anesthesia, left paratracheal pressure exhibited superior success rates during initial attempts at inserting a laryngeal mask airway (LMA), with greater ease of insertion and fewer complications [16]. These findings underscore that paratracheal pressure minimizes airway obstruction risks and does not complicate endoscopic procedures conducted by gastroenterologists.

Another study revealed that paratracheal pressure significantly reduces the risk of gastric insufflation during mask ventilation with positive pressure, where no gastric insufflation was observed in the paratracheal pressure group [7]. Additionally, mask ventilation was easier to perform, and peak inspiratory pressure increased less during mechanical ventilation [7]. In obese patients, paratracheal pressure has shown substantial benefits, markedly improving mask ventilation efficiency, increasing expiratory tidal volumes and peak inspiratory pressures without hypoxemia [17]. This suggests that paratracheal pressure could enhance mask ventilation outcomes in obese patients. Collectively, these observations indicate that paratracheal pressure offers potential advantages in clinical interventions, particularly in high-risk patient populations such as those who are obese.

We innovatively propose the application of paratracheal pressure during endoscopic iodine staining of the esophageal mucosa to mitigate coughing and pharyngeal discomfort in patients. China accounts for nearly half of the global burden of esophageal cancer and remains one of the countries with the highest mortality rates from this disease. The prognosis of esophageal cancer is closely tied to early detection and treatment: patients with advanced disease have a 5-year survival rate below 10%, while those diagnosed at an early stage and receiving timely intervention can achieve survival rates above 90% [1, 2].

Iodine staining plays a pivotal role in identifying esophageal lesions. This technique relies on the high glycogen content in normal squamous epithelial cells, which binds avidly to iodine, resulting in a characteristic dark brown discoloration of healthy mucosa. In contrast, areas deficient in glycogen—such as dysplastic or malignant lesions—fail to stain and appear yellow or pale pink. This differential staining enhances the precision of targeted biopsies [18]. Iodine chromoendoscopy is simple, cost-effective, and particularly valuable for early cancer screening in resource-limited and primary care settings. However, under general anesthesia, protective airway reflexes are suppressed, increasing the risk and clinical impact of coughing during staining. Uncontrolled coughing may lead to airway trauma, gastroesophageal reflux, and potential aspiration, posing significant safety concerns [19, 20].

Paratracheal pressure not only reduces intraoperative coughing but also alleviates pharyngeal irritation caused by iodine reflux. By compressing the cervical esophagus—particularly at the left supraclavicular region—the lumen is mechanically occluded, limiting distal flow of iodine and minimizing accumulation in the proximal esophagus and hypopharynx. Given that the upper esophagus is anatomically close to the larynx and richly innervated by vagal branches (e.g., recurrent laryngeal nerves), direct stimulation of this area can trigger strong reflex coughing. Paratracheal pressure attenuates local mucosal irritation and suppresses vagally mediated hypersensitive responses, thereby reducing the incidence of cough reflexes.

The paratracheal pressure group showed a significant decrease in MAP and HR following the procedure. Coughing is a strong physiological reflex that triggers activation of the sympathetic nervous system, resulting in increased HR and elevated MAP. By reducing coughing, paratracheal pressure mitigates sympathetic activation and promotes hemodynamic stability. Additionally, reduced stimulation of the pharyngolaryngeal region diminishes vagally mediated bradycardia or blood pressure fluctuations, thereby minimizing abrupt shifts between sympathetic and parasympathetic tone and contributing to more stable cardiovascular dynamics. Collectively, these findings support paratracheal pressure as a safe, effective, and reliable adjunct during endoscopic iodine staining, particularly in high-risk scenarios such as sedated or anesthetized patients. Its potential benefits in improving patient comfort, reducing airway complications, and enhancing procedural stability warrant broader clinical adoption and further investigation. Furthermore, emerging evidence suggests that paratracheal pressure is effective in various other clinical settings. For instance, Thappa et al. reported its successful use in patients with undiagnosed neck vascular masses, where it helped prevent aspiration and contributed to improved clinical outcomes [21]. These findings underscore the broad applicability and efficacy of paratracheal pressure across diverse clinical scenarios, lending further support to our conclusion that it is a valuable technique for airway management. This prospective randomized controlled trial provides high-quality evidence that paratracheal pressure is effective and safe during endoscopic iodine staining. It offers a simple, non-invasive, and cost-effective strategy to reduce coughing and pharyngeal discomfort without requiring additional equipment or increasing procedural complexity. Given its ease of implementation, this technique holds high potential for widespread clinical adoption, particularly in resource-limited or challenging healthcare environments where low-cost, high-impact interventions are urgently needed.

Despite these promising results, several limitations should be acknowledged. First, the study was conducted at a single center with a total sample size of 80 patients, which may limit statistical power and restrict generalizability. Second, blinding was partial: although patients and some outcome assessors were blinded, the primary outcome (moderate-to-severe coughing) was evaluated by the anesthesiologist who performed the paratracheal pressure due to the brief procedural timeframe and the real-time nature of the assessment. Although standardized criteria were used, this lack of full assessor blinding may introduce observer bias. Third, although clinical outcomes were prospectively assessed, we did not employ objective measures of gastroesophageal reflux, such as 24-hour pH-impedance monitoring or esophageal manometry; thus, the direct impact of paratracheal pressure on reflux dynamics remains inferential. Fourth, while no adverse events were reported during the procedure, we did not systematically evaluate compression-related complications, including carotid artery compression, vagal or recurrent laryngeal nerve stimulation, or tracheal deviation. Future studies should incorporate structured safety monitoring to fully characterize the risk profile of this maneuver. Finally, the efficacy and safety of paratracheal pressure in specific high-risk subgroups—such as elderly patients or those with cervical spine disorders, vascular disease, or esophageal pathology—remain unclear and warrant targeted investigation. Notably, although previous studies support the anatomical plausibility of esophageal compression under paratracheal pressure, our study did not perform real-time ultrasound in any patient; thus, the mechanical effect remains indirect in our cohort. Future multicenter, large-sample, and more rigorously designed validation trials are warranted.

## Conclusions

Anesthesiologist-assisted paratracheal pressure effectively reduces the incidence of moderate-to-severe coughing and pharyngeal discomfort during endoscopic iodine staining, while also promoting greater hemodynamic stability.

## Supplementary Information

Below is the link to the electronic supplementary material.

Supplementary Material 1.

Supplementary Material 2.

Supplementary Material 3.

Supplementary Material 4.

Supplementary Material 5.

Supplementary Material 6.

Supplementary Material 7.

Supplementary Material 8.

Supplementary Material 9.

Supplementary Material 10.

Supplementary Material 11.

## Data Availability

The data supporting the findings of this study are available within the article and its supplementary materials. Additional information or requests for the datasets may be directed to the corresponding author upon reasonable request.

## Abbreviations

MAP: Mean arterial pressure
HR: Heart rate
RCT: Randomized controlled trial
BMI: Body mass index
ASA: American Society of Anesthesiologists
NIBP: Non-invasive blood pressure
ECG: Electrocardiography
SpO_2_: Peripheral oxygen saturation
SD: Standard deviation
IQR: Interquartile range
RR: Risk ratio
RD: Risk difference
OR: Odds ratio
MD: Mean difference
95% CI: 95% confidence interval
ANCOVA: Analysis of covariance
LMA: Laryngeal mask airway

## References

[1] Bray F, Laversanne M, Sung H, Ferlay J, Siegel RL, Soerjomataram I, Jemal A. Global cancer statistics 2022: GLOBOCAN estimates of incidence and mortality worldwide for 36 cancers in 185 countries. CA Cancer J Clin. 2024;74(3):229–63. 38572751 10.3322/caac.21834

[2] Liang H, Fan JH, Qiao YL. Epidemiology, etiology, and prevention of esophageal squamous cell carcinoma in China. Cancer Biol Med. 2017;14(1):33-41. 10.20892/j.issn.2095-3941.2016.0093. 10.20892/j.issn.2095-3941.2016.0093 PMC5365188 28443201

[3] Lijuan YSZSH. The diagnostic value of iodine staining and narrow - band imaging in early esophageal cancer screening. Anhui Med J. 2020;41(11):1302–5.

[4] Fan X, Gong M, Yu H, Yang H, Wang S, Wang R. Propofol enhances stem-like properties of glioma via GABA(A)R-dependent Src modulation of ZDHHC5-EZH2 palmitoylation mechanism. Stem Cell Res Ther. 2022;13(1):398. 35927718 10.1186/s13287-022-03087-5 PMC9351178

[5] Andruszkiewicz P, Wojtczak J, Wroblewski L, Kaczor M, Sobczyk D, Kowalik I. Ultrasound evaluation of the impact of cricoid pressure versus novel ‘paralaryngeal pressure’ on anteroposterior oesophageal diameter. Anaesthesia. 2016;71(9):1024–9. 27523050 10.1111/anae.13518

[6] Kim H, Chang JE, Won D, Lee JM, Kim TK, Kim MJ, Min SW, Hwang JY. Effectiveness of cricoid and paratracheal pressures in occluding the upper esophagus through induction of anesthesia and videolaryngoscopy: A Randomized, crossover study. Anesth Analg. 2022;135(5):1064–72. 35913721 10.1213/ANE.0000000000006154

[7] Gautier N, Danklou J, Brichant JF, Lopez AM, Vandepitte C, Kuroda MM, Hadzic A, Gautier PE. The effect of force applied to the left paratracheal oesophagus on air entry into the gastric antrum during positive-pressure ventilation using a facemask. Anaesthesia. 2019;74(1):22–8. 30288741 10.1111/anae.14442

[8] Fan X, Cai H, Pan B, Xie Y. Comparison of Dexmedetomidine and remifentanil on reducing coughing during emergence from anesthesia with tracheal intubation: A meta-analysis. Front Pharmacol. 2022;13:993239. 36249748 10.3389/fphar.2022.993239 PMC9561905

[9] Rolf N, Cote CJ. Frequency and severity of desaturation events during general anesthesia in children with and without upper respiratory infections. J Clin Anesth. 1992;4(3):200–3. 1610574 10.1016/0952-8180(92)90065-9

[10] Hull JH, Walsted ES, Pavitt MJ, Tidmarsh B, Selby J. An evaluation of a throat discomfort visual analogue scale in chronic cough. Eur Respir J. 2020;55(3):1901722. 10.1183/13993003.01722-2019. 10.1183/13993003.01722-2019 31744835

[11] Moonesinghe SR, Jackson AIR, Boney O, Stevenson N, Chan MTV, Cook TM, Lane-Fall M, Kalkman C, Neuman MD, Nilsson U, et al. Systematic review and consensus definitions for the standardised endpoints in perioperative medicine initiative: patient-centred outcomes. Br J Anaesth. 2019;123(5):664–70. 31493848 10.1016/j.bja.2019.07.020

[12] Francksen H, Renner J, Hanss R, Scholz J, Doerges V, Bein B. A comparison of the i-gel with the LMA-Unique in non-paralysed anaesthetised adult patients. Anaesthesia. 2009;64(10):1118–24. 19735404 10.1111/j.1365-2044.2009.06017.x

[13] Schulz KF, Altman DG, Moher D, Group C. CONSORT 2010 statement: updated guidelines for reporting parallel group randomised trials. BMJ. 2010;340:c332. 20332509 10.1136/bmj.c332 PMC2844940

[14] Schulz KF, Altman DG, Moher D, Group C. CONSORT 2010 statement: updated guidelines for reporting parallel group randomised trials. BMC Med. 2010;8:18. 20334633 10.1186/1741-7015-8-18 PMC2860339

[15] Won D, Kim H, Chang JE, Lee JM, Kim TK, Kim H, Min SW, Hwang JY. Comparison of the effects of paratracheal pressure and cricoid pressure on placement of the i-gel((R)) supraglottic airway: a randomized clinical trial. Can J Anaesth. 2024;71(7):996–1003. 38507025 10.1007/s12630-024-02741-1 PMC11266228

[16] Hur M, Lee K, Min SK, Kim JY. Left paratracheal pressure versus cricoid pressure for successful laryngeal mask airway insertion in adult patients: a randomized, non-inferiority trial. Minerva Anestesiol. 2021;87(11):1183–90. 34337919 10.23736/S0375-9393.21.15779-7

[17] Seol T, Kim H, Chang JE, Kang Y, Hwang JY. Effect of paratracheal pressure on the effectiveness of mask ventilation in obese anesthetized patients: a randomized, cross-over study. J Clin Monit Comput. 2024;38(1):31–6. 37418060 10.1007/s10877-023-01048-8

[18] Lijuan YSZSH. The diagnostic value of iodine staining and narrow - band imaging in early esophageal cancer screening. Anhui Med J. 2020;41:1302–5.

[19] Bouvet L, Barnoud S, Desgranges FP, Chassard D. Effect of body position on qualitative and quantitative ultrasound assessment of gastric fluid contents. Anaesthesia. 2019;74(7):862–7. 30963542 10.1111/anae.14664

[20] Won D, Kim H, Chang JE, Lee JM, Min SW, Ma S, Kim C, Hwang JY, Kim TK. Effect of paratracheal pressure on the glottic view during direct laryngoscopy: A randomized Double-Blind, noninferiority trial. Anesth Analg. 2021;133(2):491–9. 34081034 10.1213/ANE.0000000000005620

[21] Thappa P, Cheema K, Gowda PK, Barik AK. Paratracheal compression to prevent aspiration in a patient with an undiagnosed vascular neck mass: quick detection and improvisation - A case study. Indian J Anaesth. 2023;67(7):668–70. 37601926 10.4103/ija.ija_1000_22 PMC10436708

