# Supplementary figures and images for: Paratracheal pressure reduces the incidence of moderate-to-severe coughing during endoscopic esophageal iodine staining: a prospective, randomized controlled trial
Source: BMC Anesthesiol. 2026 Feb 2;26:152. doi: 10.1186/s12871-026-03644-y (PMC12952075; doi:10.1186/s12871-026-03644-y)

# Comparison between groups

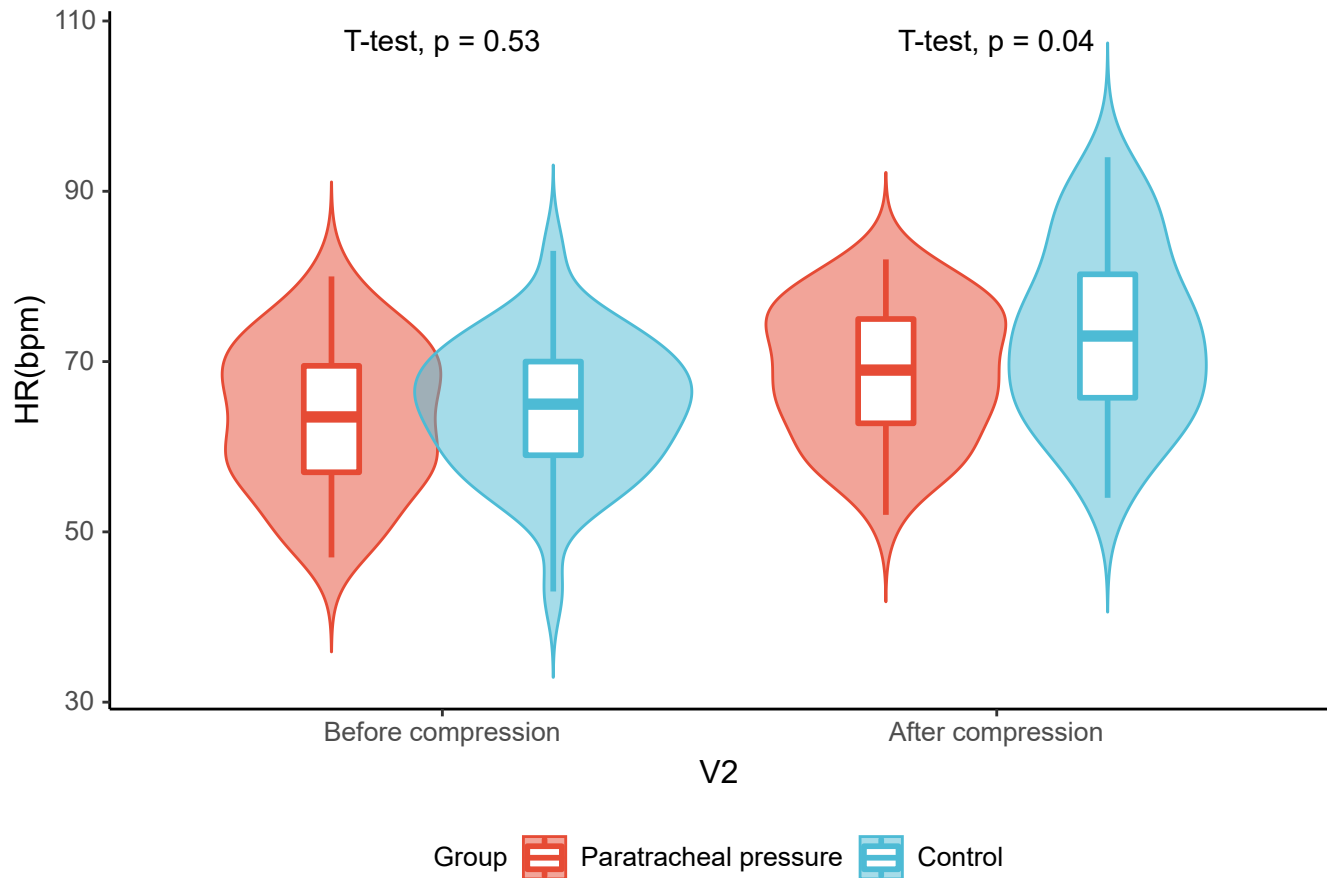

Supplement: Supplementary file 1 — Supplementary Material 1. [file 12871_2026_3644_MOESM1_ESM.pdf]

# Comparison between groups

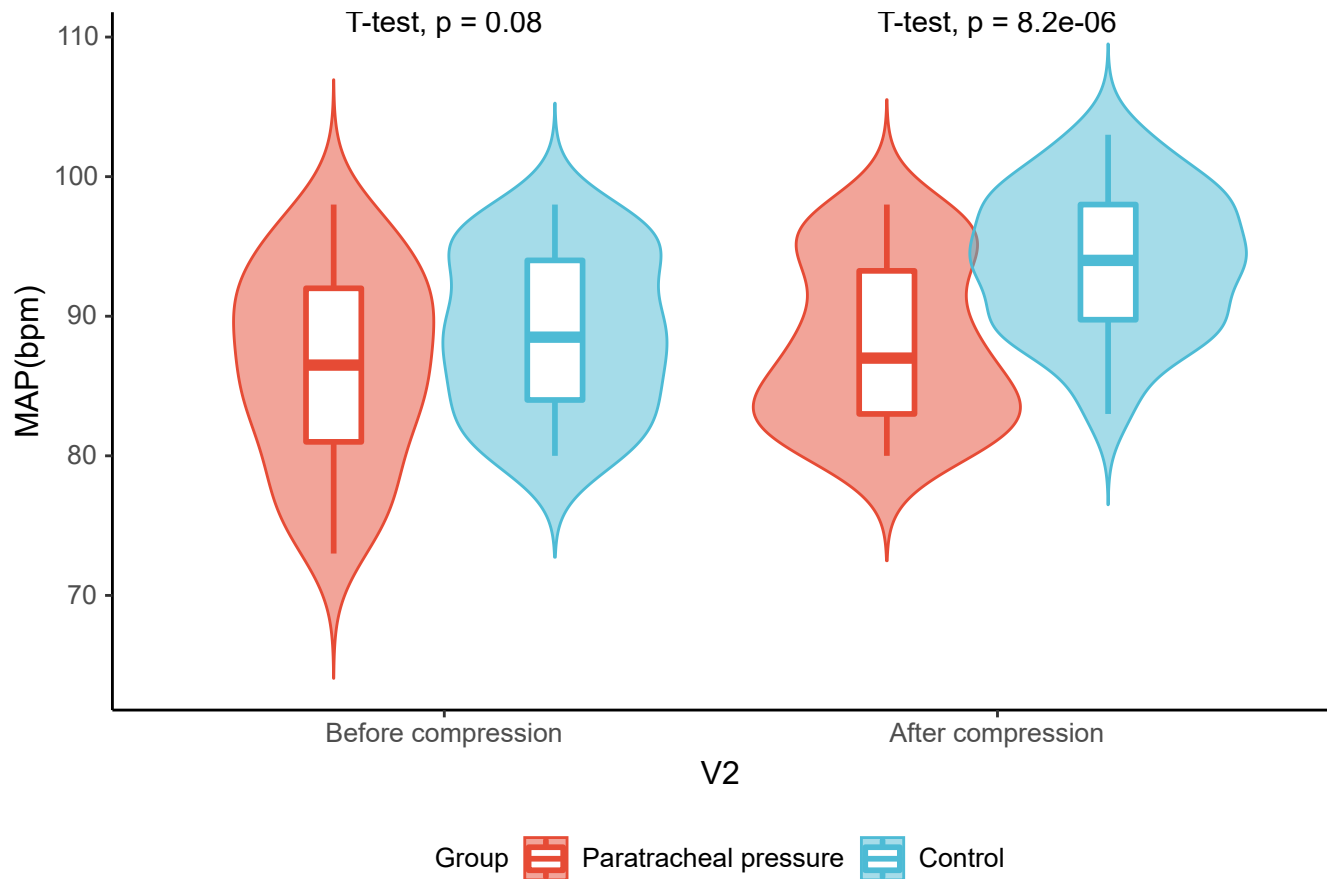

Supplement: Supplementary file 2 — Supplementary Material 2. [file 12871_2026_3644_MOESM2_ESM.pdf]

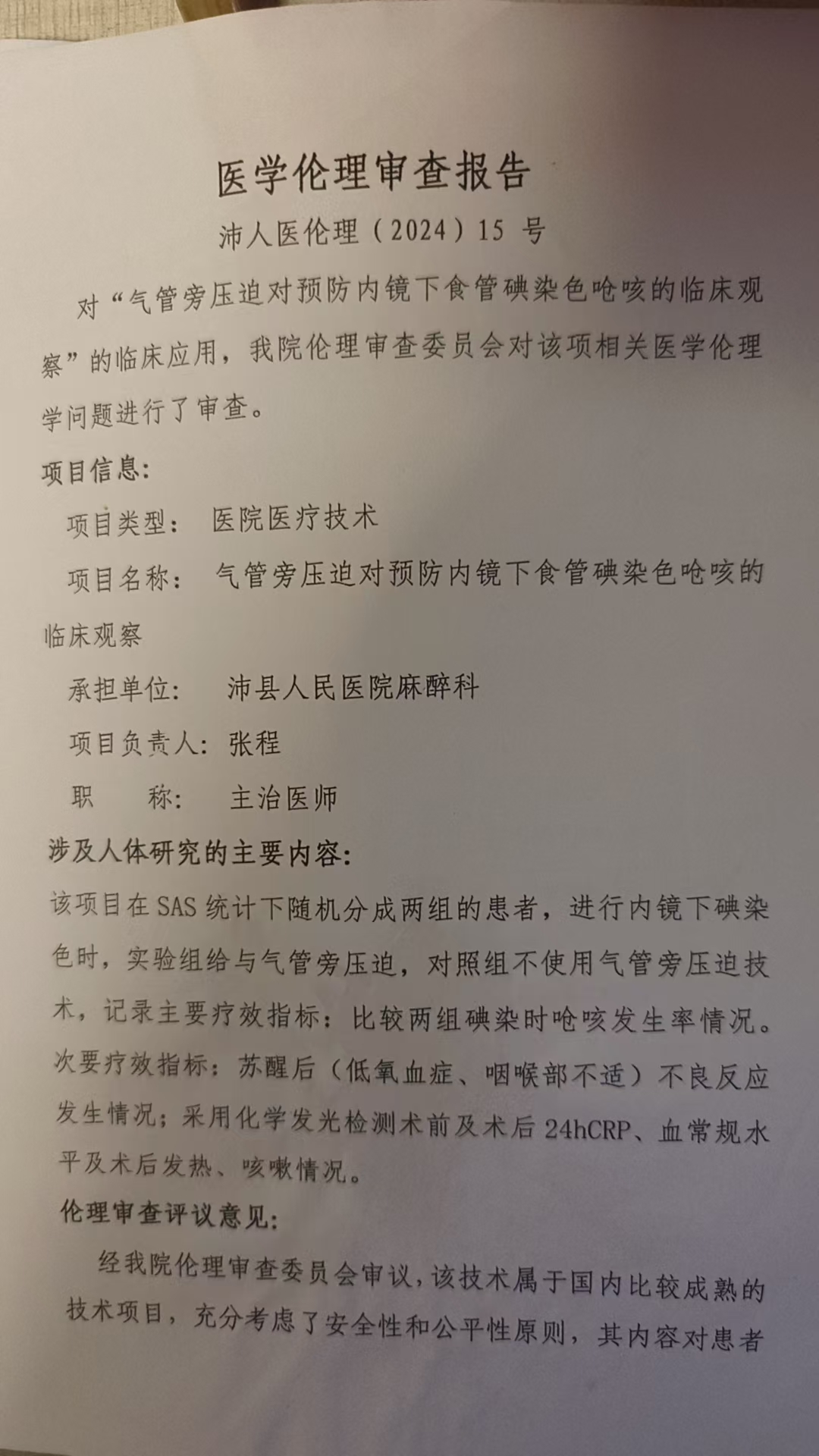

Supplement: Supplementary file 3 — Supplementary Material 3. [file 12871_2026_3644_MOESM3_ESM.jpg]

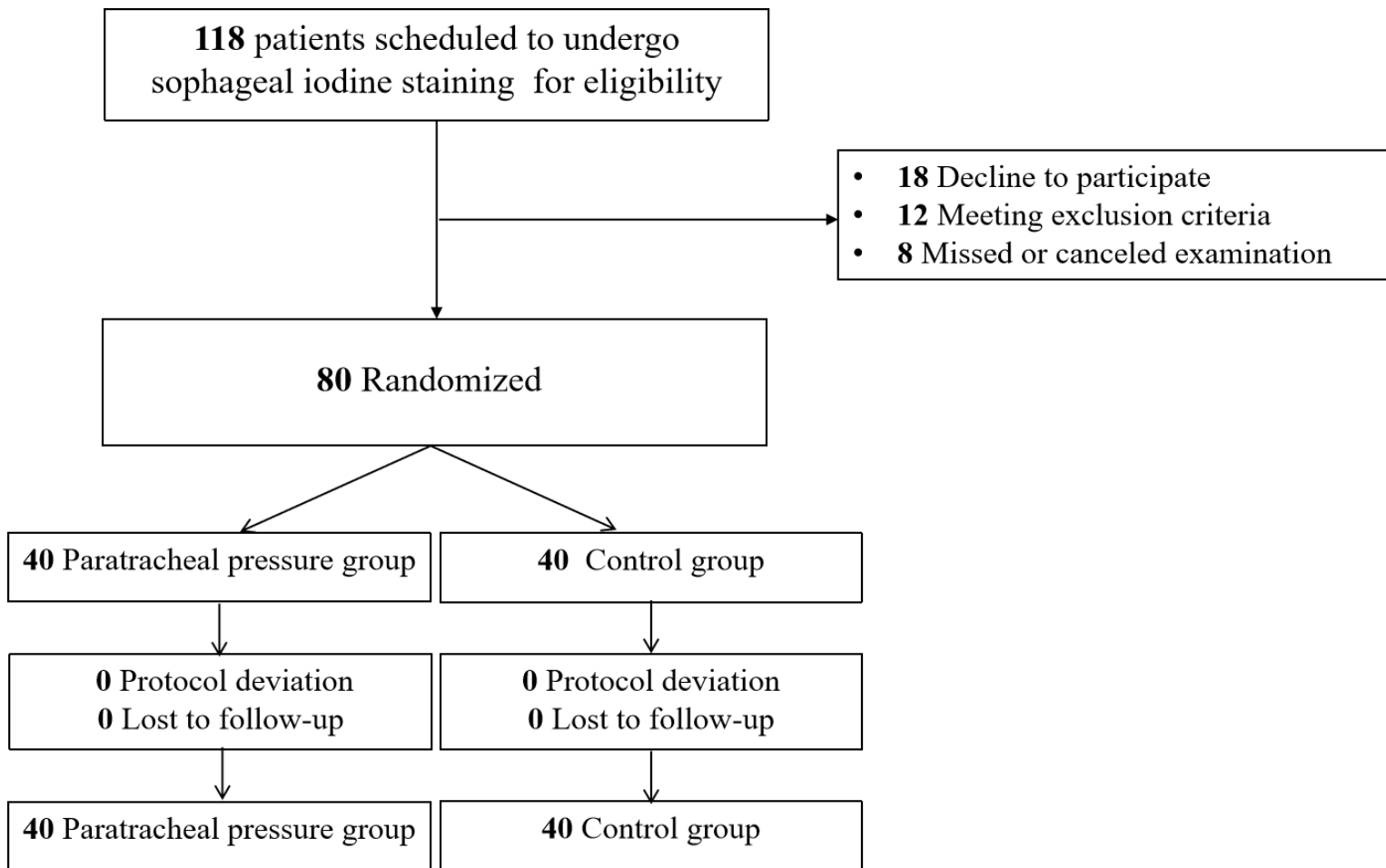

Supplement: Supplementary file 5 — Supplementary Material 5. [file 12871_2026_3644_MOESM5_ESM.pdf]

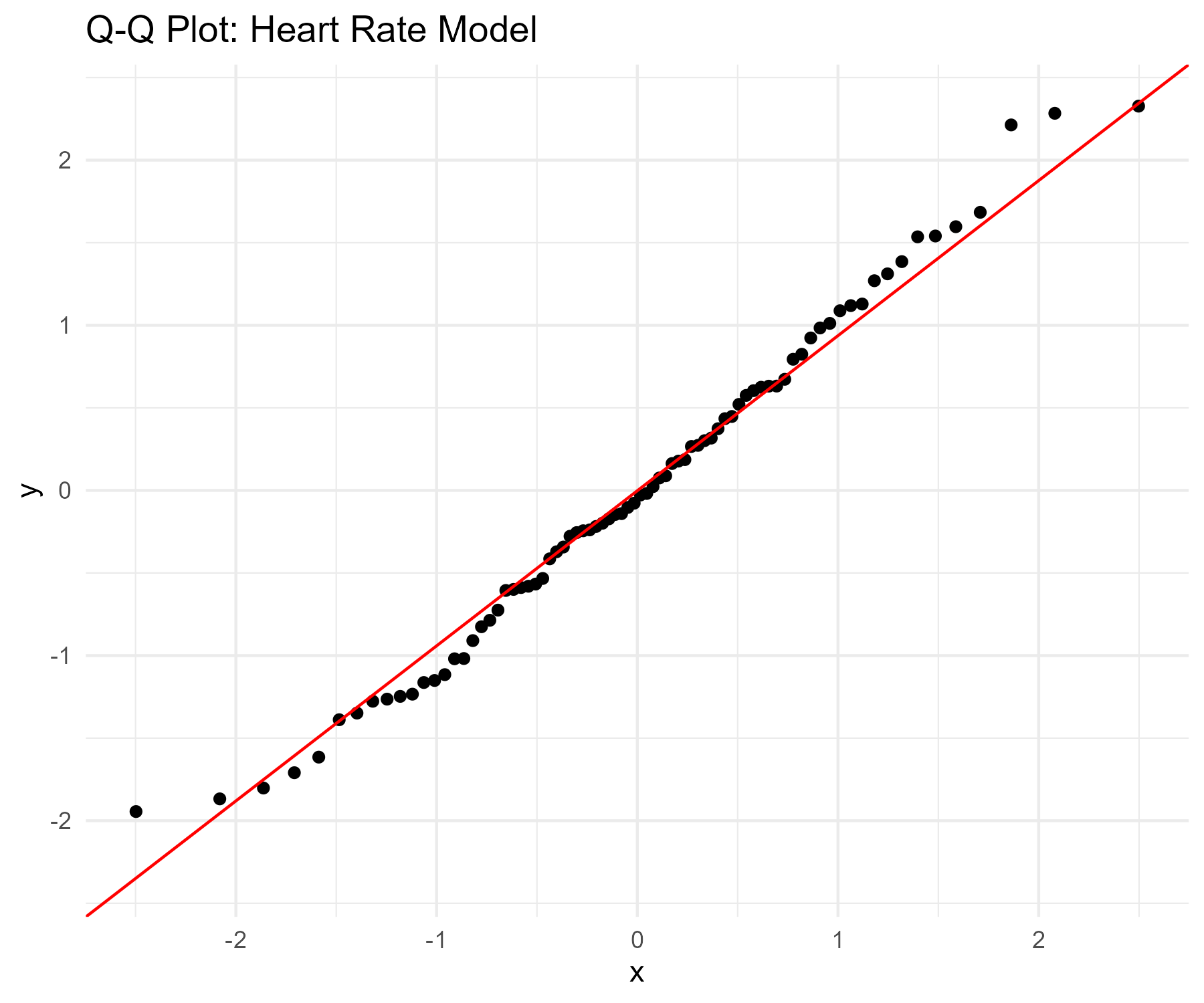

Supplement: Supplementary file 7 — Supplementary Material 7. [file 12871_2026_3644_MOESM7_ESM.png]

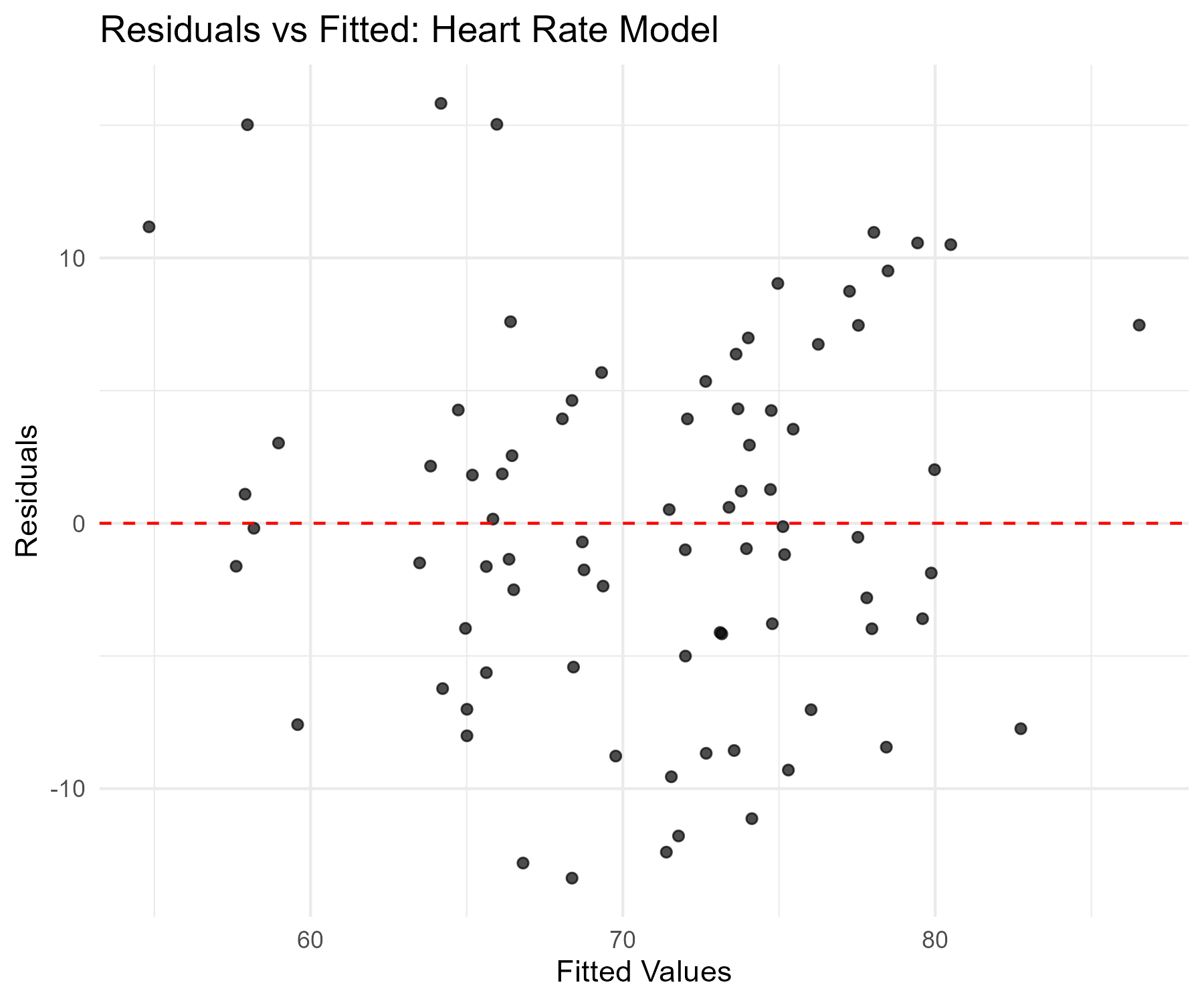

Supplement: Supplementary file 8 — Supplementary Material 8. [file 12871_2026_3644_MOESM8_ESM.png]

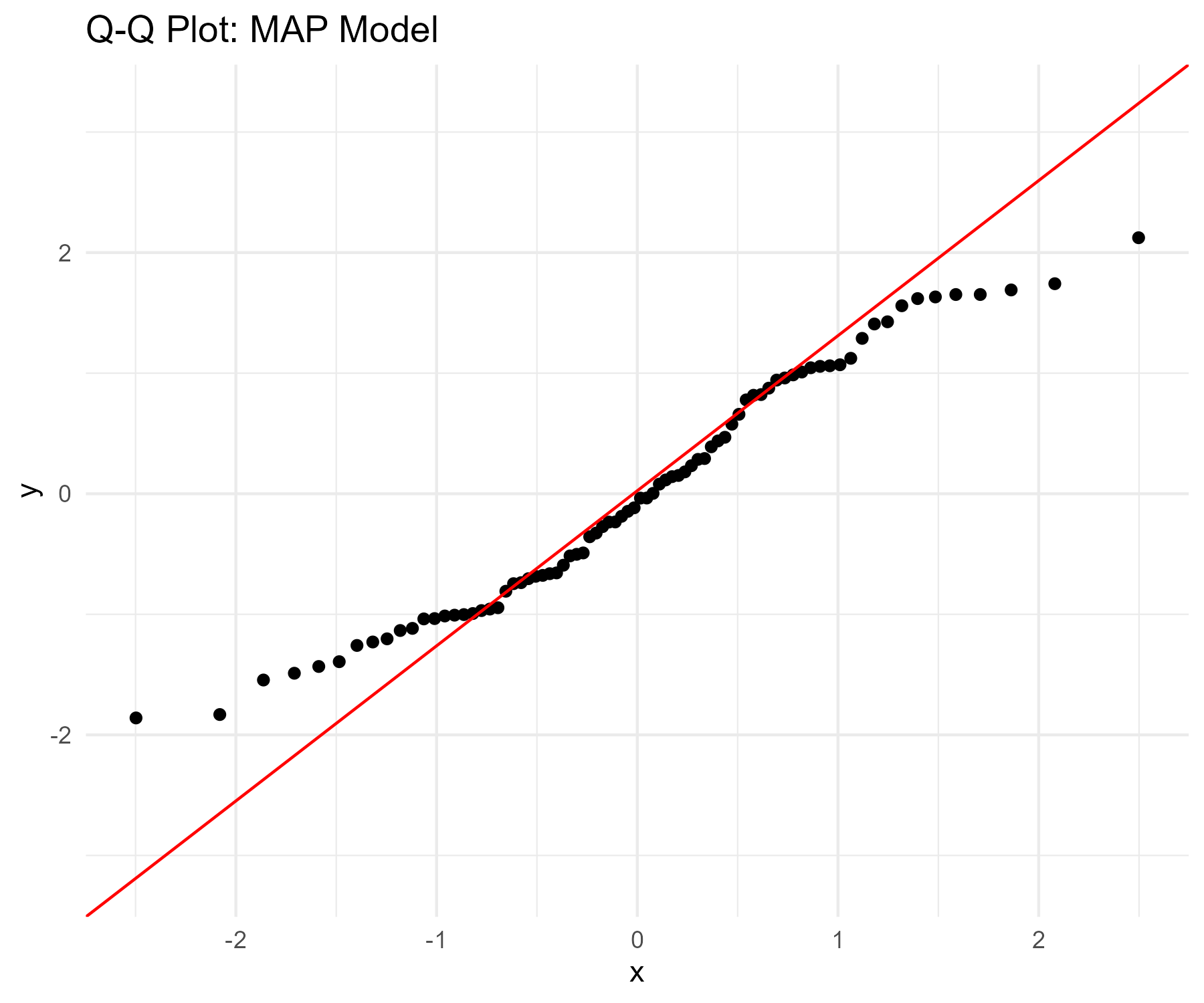

Supplement: Supplementary file 10 — Supplementary Material 10. [file 12871_2026_3644_MOESM10_ESM.png]

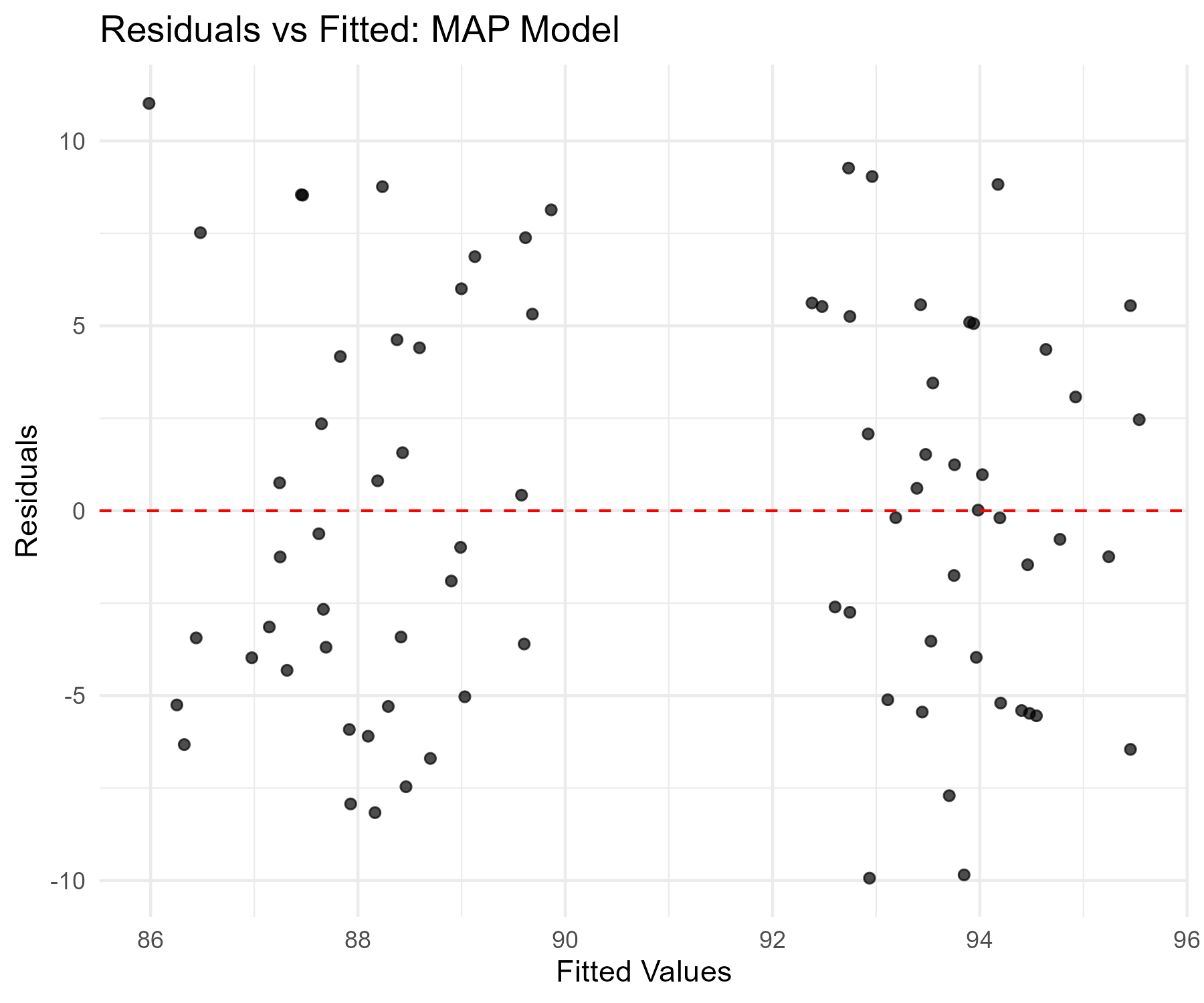

Supplement: Supplementary file 11 — Supplementary Material 11. [file 12871_2026_3644_MOESM11_ESM.png]
